# Comparison of Antimicrobial Efficacy of Ocimum sanctum (Tulsi) Extract and Chlorhexidine Against Tannerella forsythia: An In Vitro Study

**DOI:** 10.7759/cureus.72167

**Published:** 2024-10-22

**Authors:** Mithra R, Kalaivani V, Gayathri K, Ravishankar PL, Divya Vinayachandran, Sibyl Siluvai, Rajakumar S, Viola Esther

**Affiliations:** 1 Periodontology, Sri Ramaswamy Memorial (SRM) Kattankulathur Dental College and Hospital, Sri Ramaswamy Memorial Institute of Science and Technology (SRMIST), Chennai, IND; 2 Oral Medicine and Radiology, Sri Ramaswamy Memorial (SRM) Kattankulathur Dental College and Hospital, Sri Ramaswamy Memorial Institute of Science and Technology (SRMIST), Chennai, IND; 3 Public Health Dentistry, Sri Ramaswamy Memorial (SRM) Kattankulathur Dental College and Hospital, Sri Ramaswamy Memorial Institute of Science and Technology (SRMIST), Chennai, IND; 4 Pediatric and Preventive Dentistry, Sri Ramaswamy Memorial (SRM) Kattankulathur Dental College and Hospital, Sri Ramaswamy Memorial Institute of Science and Technology (SRMIST), Chennai, IND

**Keywords:** antimicrobial activity, natural products, ocimum sanctum, periodontal diseases, tannerella forsythia

## Abstract

Context: Periodontitis, a chronic inflammatory disease affecting the periodontium, is primarily caused by gram-negative anaerobic bacteria. Conventional periodontal therapy aims to eradicate pathogenic microflora through mechanical debridement, often supplemented with chemical agents. However, the use of these chemical adjuvants is frequently associated with adverse drug reactions and the development of antimicrobial resistance. Consequently, researchers are increasingly exploring herbal antibacterial agents, such as *Ocimum sanctum* (Tulsi), as viable alternatives due to their broad-spectrum antimicrobial properties and reduced side effect profile.

Aim: This study aims to analyze the antibacterial efficacy of the ethanolic extract of *O. sanctum* (Tulsi) against *Tannerella forsythia*, a key bacterium of the red complex, using chlorhexidine as a reference standard. Traditionally, chlorhexidine has been utilized as an adjunct to nonsurgical therapy in the treatment of patients with periodontal disease.

Materials and methods: The cold extraction method was used to prepare the ethanolic extract of *O. sanctum* leaves. The resultant substance was mixed into five different concentrations (3.13%, 6.25%, 12.5%, 25%, and 50%) using dimethylformamide as the solvent. The antimicrobial activity of these varying concentrations was assessed against *T. forsythia* in culture plates employing the agar well diffusion method, with 0.2% chlorhexidine serving as the positive reference. The zone of inhibition of bacterial growth has been determined and compared with that of 0.2% chlorhexidine.

Results: The ethanolic extract of *O. sanctum* exhibited highly statistically significant antibacterial activity against *T. forsythia* (p < 0.001). At a dosage of 25 mg/mL, the extract created a zone of inhibition measuring 17 mm, while at 50 mg/mL, the zone of inhibition increased to 25 mm. However, when compared to 0.2% chlorhexidine, which had a zone of inhibition of 32 mm, *O. sanctum* showed a slightly smaller inhibitory effect.

Conclusion: The extract of *Tulsi* showed notable antibacterial activity against *T. forsythia*, with the antibacterial impact observed at the 50 mg/mL dilution.

## Introduction

Periodontitis and periodontal diseases are significant oral health disorders that impact systemic health and are considered global illnesses. Periodontitis is an inflammatory disease initiated and perpetuated by dental plaque microflora, including both aerobic and anaerobic bacteria, which affect the supporting structures of the teeth. The oral cavity hosts approximately 10^14^ microbial cells and 700 distinct bacterial species. There are 1.3-10 times as many bacteria in the human body as there are human cells [1].

The plaque biofilm consists of five complexes of microorganisms from different species. Among these, *Aggregatibacter actinomycetemcomitans* is implicated in the development of active periodontal lesions [2], whereas *Tannerella forsythia*, *Prevotella intermedia*, *Porphyromonas gingivalis*, and *Treponema denticola* are linked with chronic periodontitis [3]. *P. gingivalis* also plays a role in locally aggressive periodontitis [4-6], and *P. intermedia* is involved in necrotizing ulcerative gingivitis [7].

*P. gingivalis*, *T. forsythia*, and *T. denticola* are considered the most pathogenic microbial red complex bacteria. These red complex bacteria have a role in the initial phase of biofilm formation [8,9]. These bacteria express virulence factors that can influence infection pathways within the subgingival pocket, disrupt host immune responses, and cause dysbiosis [10]. They have an association with clinical findings of periodontal disease like pocket depth and bleeding on probing.

The primary strategies for preventing and treating periodontal diseases involve reducing the microbial load (including gram-negative and gram-positive anaerobes, aerobes, viruses, fungi, and yeasts) in the oral cavity and periodontal pocket. Antimicrobial agents are used as adjuvants to conventional therapy (scaling and root planing) in periodontal therapy.

Chlorhexidine is the most common chemotherapeutic antibacterial and antiplaque agent used in the treatment of periodontitis. However, it has several adverse effects, such as altering taste and discoloring teeth [11]. Therefore, there is a need to find alternative antibacterial agents that are as effective as chlorhexidine. The antibacterial efficacy of various drugs has been investigated, using chlorhexidine as a positive control.

Phytomedicine, or plant extracts, has been researched for its effectiveness against several periodontal infections to formulate antibacterial agents. Herbal medicine has been recommended for treating a wide range of ailments in countries such as India, China, Egypt, and Greece [12]. *O. sanctum* L., commonly known as Tulsi, is a member of the Lamiaceae family and is used as a fundamental aromatic herb in Indian Ayurveda. Another name for Tulsi is “queen of the herbs” because of its moral and therapeutic properties. Tulsi is known for its ability to inhibit cyclooxygenase-2, protect against radiation, and prevent cataracts; in addition, it possesses antioxidant, anti-hyperlipidemic, cardioprotective, hepatoprotective, analgesic, antipyretic, anxiolytic, antidepressant, and immune system-enhancing properties. Tulsi is used to treat various systemic illnesses, including upper respiratory infections, bronchitis, skin diseases, and malaria [13].

Additionally, it has antimicrobial activity against a range of gram-positive and gram-negative pathogens, including Enterobacteriaceae, *Bacillus subtilis*, *Bacillus anthracis*, *Pseudomonas aeruginosa*, *Salmonella typhi*, *Salmonella pullorum*, *Salmonella newport*, *Klebsiella pneumonia*, *Proteus*, *Mycobacterium tuberculosis*, *Micrococcus pyogenes*, and *Vibrio cholerae *[14]. Furthermore, Tulsi demonstrated antifungal efficacy against *Aspergillus niger* and *Candida albicans* [15]. Tulsi functions as a potent antiviral agent against hepatitis B virus (adenovirus), coxsackievirus B1, enterovirus 71 (RNA virus), and herpes viruses (DNA virus) [16].

Bioactive components present in *O. sanctum* are phenolic compounds (antioxidants), which comprise 71% eugenol, 20% methyl eugenol, flavonoids, orientin, vicenin, and 0.7% volatile oil [13].

Despite the traditional use of Tulsi for various medicinal purposes, there is a paucity of literature documenting its antibacterial efficacy against periodontal pathogens. This study aimed to thoroughly characterize the antibacterial activity of Tulsi, by measuring the inhibitory zones using the agar well diffusion method against *T. forsythia*, a bacterium frequently linked with periodontitis. The results were contrasted with chlorhexidine, a positive control commonly employed as an adjuvant to nonsurgical therapy in the treatment of periodontitis.

## Materials and methods

The study was conducted as a laboratory investigation between March and June 2024. Extract preparation was done at Avigen Biotech Private Limited, Chromepet, Chennai, India. Microbial analysis was performed at Arihant Superspeciality Hospital, Belgaum, Karnataka, India. Ethical clearance was received from the Institutional Ethics Committee (SRMIEC: ST0324-981).

Collection and preparation of leaf extracts

*O. sanctum* leaves were procured from a local herbal shop in Perungalathur, Chennai, India. The samples were authenticated by a pharmacognosist and a botanist. The leaves were dried for 12 hours in a hot air oven at 55 °C and then powdered. The powdered leaves were extracted using 70% ethanol through cold percolation for 48 hours. Fifty grams of the powdered extract was mixed in 250 mL of ethanol. After 48 hours of evaporation, the residue was stored at 4 °C. For the assay, 1 mL of this residue was mixed with 1 mL of sterile phosphate-buffered saline (pH 7.2) to achieve a 50% concentration. Similarly, concentrations of 3.13%, 6.25%, 12.5%, 25%, and 50% were prepared by diluting the extract with dimethylformamide solvent.

Microbiological assay

In the laboratory, the agar well diffusion method was employed to identify the antibacterial effect of *O. sanctum*. *T. forsythia* colonies were cultured on blood agar plates, and their turbidity was adjusted to match a 0.5 McFarland standard. A cotton swab was used to eliminate any extra inoculum within 15 minutes. To ensure even distribution, the plates were turned 60° in between streaks. To create the six wells above the inoculated plates, a 5-mm-diameter hollow tube was heated and pressed for positive control and varying concentrations (3.13%, 6.25%, 12.5%, 25%, and 50%) of *O. sanctum*. The study involved inoculating bacteria with varying amounts of Tulsi extract on three agar plates. Each well received 50 µL of the predetermined substance assigned to it. The plates were incubated at room temperature for 48 hours in an anaerobic jar (McIntosh and Fildes). Vernier calipers were used to measure zones of inhibition. The procedure was repeated three times to obtain the corresponding inhibitory zones for the respective quantities of Tulsi extract and 0.2% chlorhexidine [17].

## Results

**Figure 1:**
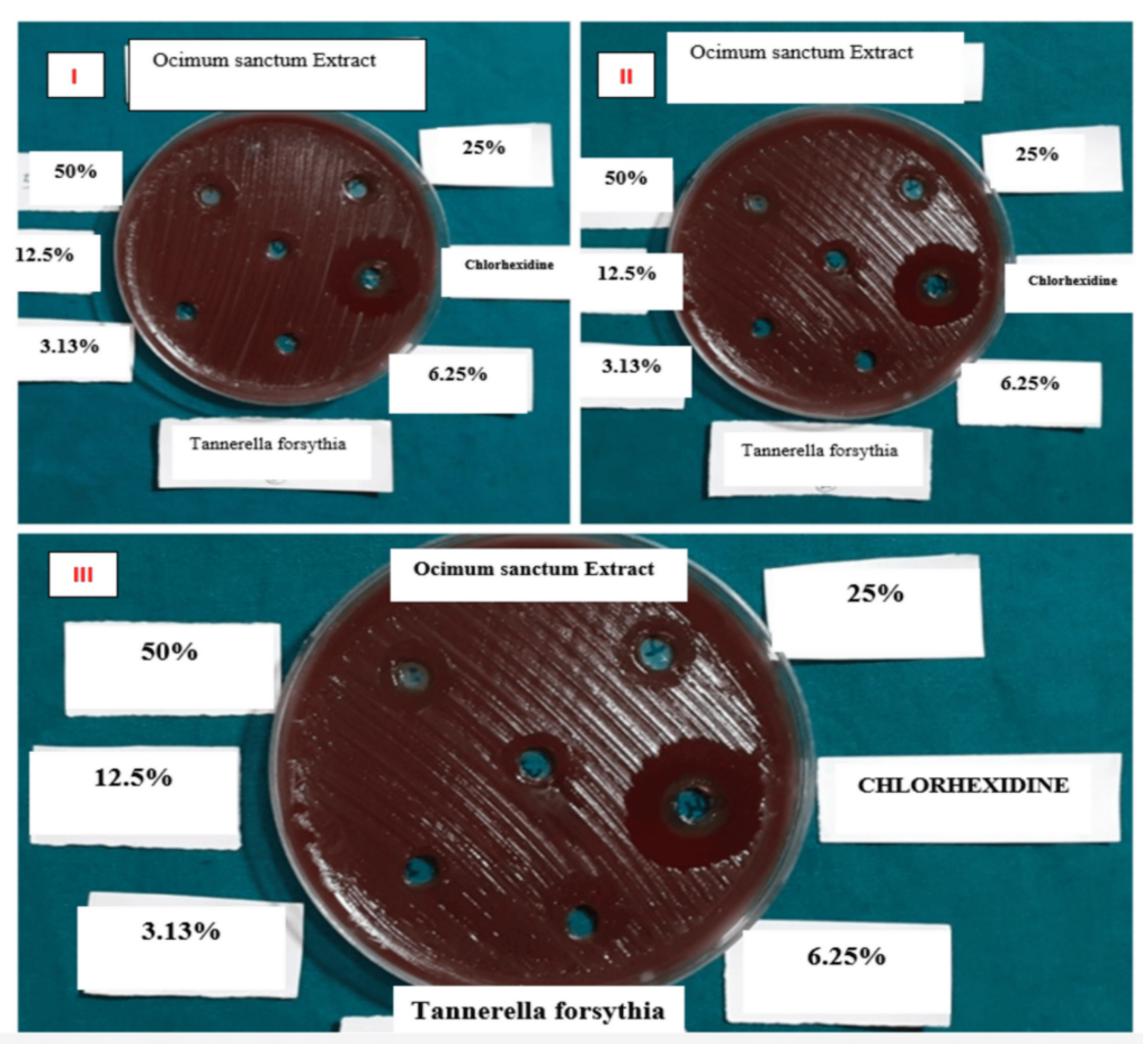
Tannerella forsythia inhibition zones determined by the agar well diffusion method. The test was repeated three times for each condition: (1) 0.2% chlorhexidine, (2) OSE (3.13 mg/mL), (3) OSE (6.25 mg/mL), (4) OSE (12.5 mg/mL), (5) OSE (25 mg/mL), and (6) OSE (50 mg/mL) OSE: *Ocimum sanctum* extract.

The inhibitory zones exhibited by *O. sanctum* (Tulsi) extracts at various quantities and 0.2% chlorhexidine (positive control) against *T. forsythia* are summarized in Table 1. The Tulsi extracts demonstrated an increasing zone of inhibition with rising concentrations against *T. forsythia*. Specifically, *O. sanctum* leaf extracts at concentrations of 3.13%, 6.25%, 12.5%, and 25% exhibited minimal zones of inhibition. However, at a concentration of 50%, Tulsi extracts displayed substantially larger zones of inhibition. In contrast, *T. forsythia *showed high susceptibility to chlorhexidine, as evidenced by the wide zones of inhibition depicted in Figure 1.

**Table 1:** Inhibitory zones exhibited by Tulsi extracts at different quantities and 0.2% chlorhexidine (positive control) against Tannerella forsythia

Ocimum sanctum (Tulsi) extract	Microorganism tested Tannerella forsythia		
	I	II	III
3.13%	16 mm	14 mm	16 mm
6.25%	16 mm	14 mm	16 mm
12.5%	18 mm	16 mm	16 mm
25%	18 mm	17 mm	16 mm
50%	24 mm	25 mm	22 mm
Chlorhexidine	32 mm	30 mm	30 mm

## Discussion

Indian holy basil (*O. sanctum* (Linn.)) or Tulsi, is a widely recognized medicinal herb extensively used in India. This plant possesses a complex chemical composition, including a broad spectrum of nutrients and physiologically active compounds. The essential oils extracted from *O. sanctum* leaves contain five primary constituents: germacrene-A, eugenol, caryophyllene, caryophyllene oxide, and clemene, all of which contribute to its antibacterial properties. Additionally, the plant contains various phytochemicals such as oleanolic acid, rosmarinic acid, and ursolic acid [13].

Research by Oyedemi et al. demonstrated the diverse mechanisms through which the active ingredients in *O. sanctum* exert their effects against pathogens [18]. The study revealed that the oil constituents cause cell lysis by inducing leakage of protein and lipid contents. Furthermore, other researchers identified eugenol, alpha-terpineol, and gamma-terpinene as particularly potent in causing protein content leakage in both gram-positive and gram-negative bacteria [18].

Singhal et al. explained the antibacterial action of Tulsi extract through its ability to convert silver ions into silver nanoparticles [19]. So, it possesses antibacterial properties against both gram-positive and gram-negative bacteria. Additionally, *O. sanctum* exhibits immunomodulatory effects, enhancing the host’s defense against infections by elevating interferon, interleukin-4, and T helper cell levels [20].

The essential oils of Tulsi, primarily phenolic in nature, are believed to possess antimicrobial properties that disrupt cytoplasmic membranes and cause potassium leakage, potentially leading to cell death [21].

In this in vitro study, *O. sanctum* extracts were tested against the periodontal pathogen *T. forsythia* at various concentrations. The 25% and 50% concentrations exhibited zones of inhibition measuring 18 mm and 24 mm, respectively. However, 0.2% chlorhexidine was better than *O. sanctum* against *T. forsythia*. These findings are consistent with a study by Mallikarjun et al., which reported that *O. sanctum* extracts at 5% and 10% concentrations demonstrated inhibition zones similar to doxycycline (control) against *P. intermedia*, *A. actinomycetemcomitans*, and *P. gingivalis* using the agar well diffusion method [17]. Similarly, a study by Eswar et al. showed that O. sanctum at a 6% concentration exhibited antibacterial effects against *A. actinomycetemcomitans* [22].

Given that *T. forsythia* is a periodontal pathogen significantly associated with the onset of periodontitis, this study intended to further explore the antibacterial activity of Tulsi. Although our findings are preliminary, further research is warranted to validate the safety and efficacy of *O. sanctum* as an adjunct to conventional periodontal treatment.

In this study, only ethanol was used in the preparation of the extract. If the effects of extracts prepared using other solvents had been evaluated, it may have provided a more comprehensive understanding of the antimicrobial properties of the agent. Different solvents may extract various bioactive compounds, potentially influencing the efficacy of the extract.

To further investigate the antimicrobial effects of the agent in greater depth, more advanced methods such as time-kill assays and flow cytometric techniques are recommended. These approaches can offer more detailed insights into the kinetics and mechanisms of antimicrobial actions, providing a clearer understanding of how the agent affects microbial populations over time.

## Conclusions

Phytochemicals isolated from Tulsi (*O. sanctum*) represent promising alternatives for the prevention and management of periodontitis. These antibacterial agents offer a safe and cost-effective approach, potentially reducing the reliance on conventional antibiotics and mitigating issues related to drug resistance and adverse side effects. Further research is warranted to elucidate their mechanisms of action and to validate their clinical efficacy.
